# Determinants and reference values of the 6-min walk distance in the general population—results of the population-based STAAB cohort study

**DOI:** 10.1007/s00392-023-02373-3

**Published:** 2024-01-18

**Authors:** Caroline Morbach, Nicola Moser, Vladimir Cejka, Michael Stach, Floran Sahiti, Fabian Kerwagen, Stefan Frantz, Rüdiger Pryss, Götz Gelbrich, Peter U. Heuschmann, Stefan Störk, on behalf of the STAAB consortium, C. Maack, G. Ertl, M. Fassnacht, C. Wanner, R. Leyh, J. Volkmann, J. Deckert, H. Faller, R. Jahns

**Affiliations:** 1 Department Clinical Research & Epidemiology, Comprehensive Heart Failure Center, University and University Hospital Würzburg, Am Schwarzenberg 15, 97078 Würzburg, Germany; 2 Department Internal Medicine I, University Hospital Würzburg, Am Schwarzenberg 15, 97078 Würzburg, Germany; 3 Institute of Clinical Epidemiology and Biometry, University of Würzburg, Würzburg, Germany; 4 Clinical Trial Center, University Hospital Würzburg, Würzburg, Germany

**Keywords:** Six-minute walk test, Physical fitness, Functional capacity, Cardiopulmonary exercise capacity, Normal values, Online calculator

## Abstract

**Aims:**

The 6-min walk test is an inexpensive, safe, and easy tool to assess functional capacity in patients with cardiopulmonary diseases including heart failure (HF). There is a lack of reference values, which are a prerequisite for the interpretation of test results in patients. Furthermore, determinants independent of the respective disease need to be considered when interpreting the 6-min walk distance (6MWD).

**Methods:**

The prospective *Characteristics and Course of Heart Failure Stages A-B and Determinants of Progression* (STAAB) cohort study investigates a representative sample of residents of the City of Würzburg, Germany, aged 30 to 79 years, without a history of HF. Participants underwent detailed clinical and echocardiographic phenotyping as well as a standardized assessment of the 6MWD using a 15-m hallway.

**Results:**

In a sample of 2762 participants (51% women, mean age 58 ± 11 years), we identified age and height, but not sex, as determinants of the 6MWD. While a worse metabolic profile showed a negative association with the 6MWD, a better systolic and diastolic function showed a positive association with 6MWD. From a subgroup of 681 individuals without any cardiovascular risk factors (60% women, mean age 52 ± 10 years), we computed age- and height-specific reference percentiles.

**Conclusion:**

In a representative sample of the general population free from HF, we identified determinants of the 6MWD implying objective physical fitness associated with metabolic health as well as with cardiac structure and function. Furthermore, we derived reference percentiles applicable when using a 15-m hallway.

**Graphical abstract:**

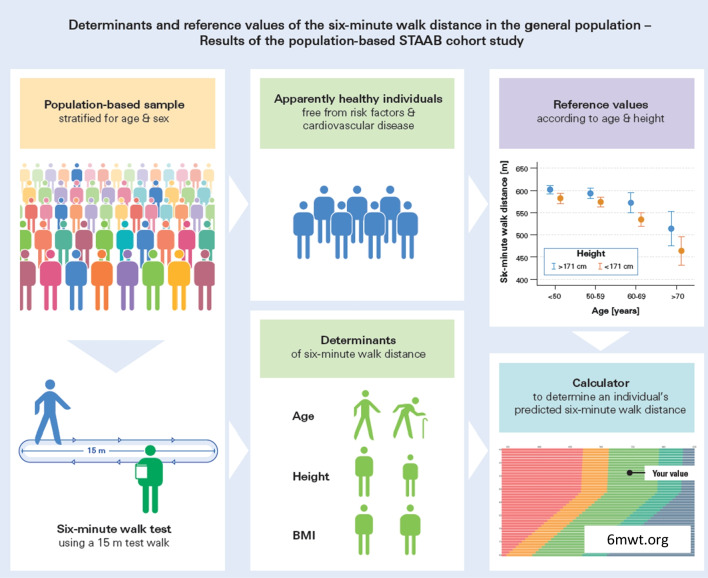

**Supplementary Information:**

The online version contains supplementary material available at 10.1007/s00392-023-02373-3.

## Introduction

The six-minute walk test (6MWT) reports the distance a subject is able to cover within a time span of 6 min (6MWD). As such, the 6MWT is a simple, safe, and inexpensive tool to estimate the functional capacity of an individual. Developed in the 1960s for the assessment of soldiers [1], the test was later refined for the application in patients with chronic bronchitis [2]. Nowadays, the 6MWT is frequently used to assess the functional capacity of patients with chronic respiratory diseases, [3, 4] heart failure, [5–7] infectious diseases, [8, 9] neurological disorders, [10–13] and cancer [14]. Further, the 6MWD has become an accepted end-point in clinical trials including heart failure [5, 15].

The latest recommendations of the American Thoracic Society ascribe to the 6MWT a good construct validity and test–retest reliability, a strong relationship with exercise performance and physical activity, and favorable responsiveness to treatment effects in patients with chronic respiratory diseases [16]. In patients with heart failure and preserved ejection fraction, the 6MWD correlated with invasively derived work load corrected pulmonary capillary wedge pressure[17] and the 6MWD carries prognostic information in patients with heart failure [5, 18–20]. The 6MWT is sensitive to variations in methodology including track length, oxygen supplementation, and encouragement; hence, stable conditions are key to ensure comparability of test results [5, 16] and walking courses of 25–30-m length are recommended [5, 16, 17].

Nevertheless, the recommendation to use a walking course of 25–30-m length is challenging to provide in clinical practice. Thus, mainly due to space constraints, several alternative protocols have been applied and used with comparable diagnostic quality [21–25].

Given the heterogeneity in methodology in addition to further factors potentially influencing the 6MWD like age, sex, and anthropometric characteristics, there is a lack of normal values serving as reference point for the assessment of impaired functional capacity. Reference equations were derived from several, mostly smaller study samples, but showed high heterogeneity and low predictive utility regarding the effective 6MWD [5, 16].

In the present study, we aimed to assess determinants of the 6MWD in a population-based cohort and to derive reference values from healthy individuals of a representative sample of the general population. Based on these results, we developed a calculator that also allows classifying the measured 6MWD in relation to the predicted 6MWD.

## Methods

### Study sample

The population-based *Characteristics and Course of Heart Failure Stages A-B and Determinants of Progression* (STAAB) Cohort Study recruited a representative sample of individuals without self-reported heart failure from the general population of Würzburg, Germany, between 12/2013 and 10/2017. A random sample of residents of the City of Würzburg (source population 124,297 inhabitants as of 2011 census) was drawn in November 2013 from the local registration office with predefined age and sex strata with ratios 1:1 for sex, and 10:27:27:27:10 for age groups of 30–39, 40–49, 50–59, 60–69, and 70–79 years, respectively. The detailed study design and methodology have been published [26].

All study-related procedures were subjected to a rigid and regular quality control process. The STAAB cohort study protocol and procedures comply with the Declaration of Helsinki and received positive votes from the Ethics Committee of the Medical Faculty as well as from the data protection officer of the University of Würzburg (vote #98/13). All participants provided written informed consent prior to any study examination [26].

The current analysis is based on the first follow-up examination, performed between 12/2017 and 08/2021. All participants attending the baseline examination were invited for a follow-up visit of about 3-h duration in the Joint Survey Unit of the Comprehensive Heart Failure Center, Würzburg, Germany, and gave their respective informed consent.

### Clinical assessment

The follow-up visit included blood collection (> 8 h of fasting) for routine laboratory parameters, physical examination, anthropometry, assessment of blood pressure, and an electrocardiogram according to pre-specified standards [26, 27]. Further, all participants underwent an extensive, pre-specified transthoracic echocardiography protocol (Vivid S6 or Vivid E95, GE Healthcare, Horten, Norway) performed by dedicated certified personnel that was quality-controlled on a regular basis [26–28].

### Six-minute walk test

The 6MWT was performed according to a standardized protocol using a 15-m test distance located in an undisturbed, straight, and flat indoor hallway. Each participant without contraindications (e.g., instable angina pectoris or myocardial infarction within the previous 4 weeks, blood pressure > 180/100 mmHg, or resting heart rate > 120 bpm), performed the 6MWT once, under the supervision of a trained staff member (graphical abstract). Participants were instructed to cover as much ground as possible within 6 min, without running or jogging (“walk as far as possible”). They could slow down or stop if necessary, but should resume walking as soon as possible. During the 6MWT, participants were encouraged verbally every 30 s, and the remaining time was announced every 2 min. Blood pressure, heart rate, and the Borg rating scale of perceived dyspnea [16] were assessed before and after the 6MWT. Additionally, the Borg rating scale of perceived exertion [29] was assessed after the 6MWT. The 6MWD was calculated from the number of 15-m laps completed within the 6 min plus the remaining meters of the last, incomplete lap and documented. Participants who terminated the test prematurely or were impaired due to specific reasons (e.g., inadequate footwear, musculoskeletal disorder, severe cardiac or pulmonary disease, neurological disorder) were graded as having an invalid 6MWD and were excluded from further analyses.

### Subgroup “apparently healthy”

A subgroup of individuals, who were free from known cardiovascular disease and cardiovascular risk factors including hypertension (blood pressure ≥ 140/90 mmHg or anti-hypertensive drug), smoking (current or ex-smoker), obesity (body mass index > 30 kg/m^2^), dyslipidemia (low density lipoprotein ≥ 190 mg/dL or lipid-lowering therapy), or diabetes mellitus (HbA1c > 6.5% or fasting plasma glucose > 7 mmol/L or 2 h plasma glucose > 11.1 mmol/L), were defined as “apparently healthy.” This subgroup was selected to derive reference values.

### Quality assurance

The effect of test–retest variability was evaluated based on information of healthy volunteers, who performed the 6MWT twice with at least 24 h between both tests. These volunteers additionally performed a third 6MWT (again with at least 24 h between tests) using the conventional 30-m hallway protocol allowing to compare the impact of test distance on the 6MWT result.

### Data analysis

Statistical analysis was performed using the IBM SPSS Statistics (version 28.0) software. A *p*-value < 0.05 was considered as statistically significant. Determinants of the 6MWD were identified from the total sample of individuals with valid 6MWT result applying generalized linear models. The variable age was adjusted for sex. The variable sex was first adjusted only for age, then adjusted additionally for body height. All other variables were adjusted for sex and age. Multiplicative interaction terms with the variable sex were sought. In the case of a significant interaction with sex, the effect estimates were reported separately for women and men. We present the effect estimate with its 95% confidence interval (95%-CI),* p*-value of the effect estimate, and *p*-value of the sex interaction.

From the subgroup of apparently healthy individuals, selected as described above, reference percentiles of the 6MWD were computed using a non-linear regression with a piecewise linear function. The distribution of the residuals of the 6MWD was analyzed using the Shapiro–Wilk test. We display the 2.5 to the 97.5 percentiles. Percentiles for intermediate values of age and body height can be determined by linear interpolation as exemplified in the Appendix.

## Results

**Fig. 1:**
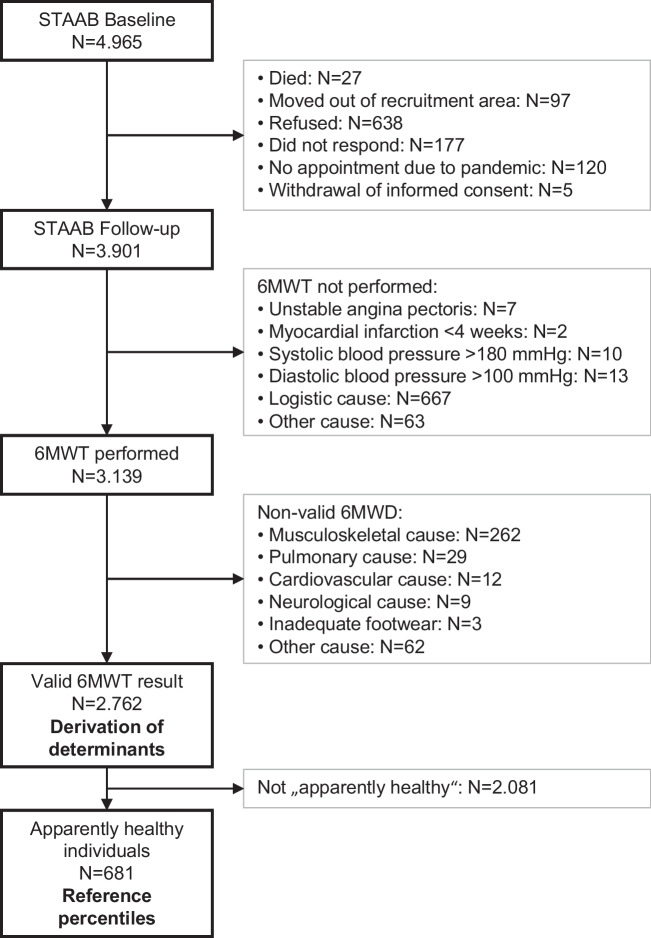
Study consort. STAAB, population-based *Characteristics and Course of Heart Failure Stages A-B and Determinants of Progression* Cohort Study; 6MWT, 6-min walk test; 6MWD, 6-min walk distance; BP, blood pressure

Of 4965 STAAB participants (52% women, 55 ± 12 years), 3901 (79%; 52% women, 58 ± 11 years) attended the first follow-up examination. Of those, 2762 (71%; 51% women, 58 ± 11 years) with valid 6MWD entered the analyses (Fig. 1). When compared to participants without valid 6MWD, those with valid 6MWD were significantly younger, less often female, and had a more favorable comorbidity and risk factor profile (Table 1).

**Table 1:** Characteristics of STAAB participants

	Total derivation sample	Subgroup without valid 6MWD	Subgroup with valid 6MWD	Subgroup “apparently healthy”
*N* (% of total sample)	3901 (100)	1139 (29)	2762 (71)	681 (25% of valid 6MWD)
Age [years]	58 (11)	60 (12)	58 (11)*	52 (10)
Female sex	2045 (52)	631 (55)	1414 (51)*	408 (60)
Body height [cm]	171 (9)	170 (9)	171 (9)*	171 (9)
Body mass index [kg/m^2^]	26.7 (5.1)	27.9 (6.0)	26.2 (4.5)*	23.8 (2.9)
Systolic blood pressure [mmHg]	130 (17)	132 (19)	130 (17)*	122 (12)
Diastolic blood pressure [mmHg]	77 (10)	77 (11)	77 (10)	74 (8)
Resting heart rate [min^−1^]	67 (10)	69 (11)	67 (10)*	66 (9)
Comorbidities				
Hypertension^**÷**^	1938 (49)	639 (56)	1299 (47)*	0 (0)
Dyslipidemia^**§**^	613 (16)	215 (19)	398 (14)*	0 (0)
Cardiovascular disease^+^	266 (7)	113 (10)	153 (6)*	0 (0)
Diabetes mellitus^**¥**^	268 (7)	103 (9)	165 (6)*	0 (0)
Obesity^#^	785 (20)	320 (28)	465 (17)*	0 (0)
Smoker** ~ **	1984 (51)	566 (50)	1418 (51)	0 (0)
Blood analysis				
eGFR [mL/min/1.73 m^2^]	81 (15)	79 (16)	82 (14)*	84 (14)
Hemoglobin [g/dL]	14.0 (1.2)	14.0 (1.2)	14.1 (1.2)*	13.8 (1.1)
LDL cholesterol [mg/dL]	117 (34)	116 (35)	117 (34)	115 (30)
HDL cholesterol [mg/dL]	62 (17)	60 (17)	62 (17)*	65 (16)
Triglycerides [mg/dL]	117 (76)	123 (72)	115 (78)*	94 (56)
HbA1c [%]	5.6 (0.6)	5.6 (0.6)	5.5 (0.5)*	5.4 (0.3)
Fasting glucose [mmol/L]	5.2 (1.0)	5.2 (1.1)	5.2 (1.0)	4.9 (0.6)
NT-proBNP [pg/mL]	64 (36; 112)	76 (42; 145)	60 (34; 103)*	52 (30; 86)
Echocardiography				
LAVi [mL/m^2^]	26 (8)	27 (9)	25 (7)*	24 (7)
LVEDVi [mL/m^2^]	48 (10)	47 (11)	49 (10)*	49 (10)
LVMi [g]	74 (18)	77 (20)	74 (17)*	69 (16)
LV stroke volume [mL]	55 (15)	53 (15)	55 (14)*	54 (14)
LV cardiac output [L/min]	3.5 (1.0)	3.5 (1.0)	3.5 (1.0)	3.4 (1.0)
LV ejection fraction [%]	59 (5)	58 (6)	59 (5)*	60 (5)
E/e’_average_	8.4 (2.7)	9.0 (3.0)	8.2 (2.6)*	7.4 (2.3)
e’_average_ [cm/s]	8.8 (2.6)	8.3 (2.5)	9.0 (2.6)*	10.2 (2.8)

Data are presented as mean (standard deviation), median (quartiles), or *n* (%), as appropriate

*eGFR* estimated glomerular filtration rate, *LDL* low-density lipoprotein, *HDL* high-density lipoprotein, *HbA1c* glycosylated hemoglobin A1c, *NT-proBNP* N-terminal pro B-type natriuretic peptide, *LAVi* left atrial volume index, *LVEDVi* left ventricular end diastolic volume index, *LVMi* left ventricular muscle mass index, *LV* left ventricular, *E* early mitral valve inflow velocity, *e'* early myocardial relaxation velocity

^÷^Hypertension: blood pressure ≥ 140/90 mmHg or taking antihypertensive pharmacotherapy

^§^Dyslipidemia: LDL cholesterol ≥ 190 mg/dL or taking a lipid-modifying drug

^+^Cardiovascular disease was self-reported and inquired the terms or respective descriptors of “cardiovascular disease,” “myocardial infarction,” “percutaneous transluminal coronary angioplasty or stent,” “peripheral arterial disease,” “stroke”

^¥^Diabetes mellitus: HbA1c > 6.5% or taking a blood glucose–lowering drug

^#^Adiposity: BMI > 30 kg/m^2^

^Smokers: active smokers or ex-smokers

*Significant difference (*p* < 0.05) between participants with and without a valid 6-min walk test result

The mean 6MWD of the total sample was 542 ± 86 m. Blood pressure before and after the 6MWT were 130 ± 17/77 ± 10 mmHg and 156 ± 22/83 ± 10 mmHg, respectively. Perceived dyspnea were 0.0 before and 1.0 immediately after the 6MWT; the perceived exertion immediately after the 6MWT was 10.0.

### Determinants of the 6MWD

The 6MWD in the 2762 participants with a valid 6MWT was significantly associated with sex, age, and body height (all *p* < 0.001), the latter two without significant interaction with sex (Table 2). After adjustment for body height and age, the association of sex with the 6MWD lost statistical significance (Table 2). Further, the 6MWD was positively associated with higher cholesterol levels, left ventricular end-diastolic volume, left ventricular ejection fraction, and left ventricular stroke volume (men only), as well as with higher left ventricular relaxation velocity, while we observed a negative association of 6MWD with higher resting heart rate, estimated glomerular filtration rate (eGFR), body mass index, trigylcerides, glycosylated hemoglobin (HbA1c), fasting glucose, and NT-proBNP, as well as with higher E/e´, a measure of left ventricular filling pressure (Table 3).

**Table 2:** Impact of age, body height, and sex on the 6-min walk distance

Variable	Effect estimate [m] (95% CI)	*p*-value (effect estimate)	*p*-value (interaction with sex)
Age [per 10 years]*	–34.0 (–36.3 to –31.7)	< 0.001	0.860
Body height [per 10 cm]**	+ 17.0 (+ 13.1 to + 21.1)	< 0.001	0.135
Sex	–17.7 (+ 12.0 to + 23.5)	< 0.001	-
Sex***	–19.3 (–24.3 to –14.3)	< 0.001	-
Sex#	+ 3.0 (–4.2 to + 10.2)	0.411	-

*Adjusted for sex

**Adjusted for sex and age

***Adjusted for age

^#^Adjusted for age and body height

Linear regression, using female sex as reference

**Table 3:** Clinical, laboratory, and echocardiographic determinants of the 6-min walk distance (effect estimates are adjusted for age and sex)

Variable	Effect estimate [m] (95% CI)	*p*-value for effect estimate	*p*-value for interaction with sex
Systolic blood pressure [per 10 mmHg]	–1.4 (–3.0 to + 0.3)	0.100	0.552
Diastolic blood pressure [per 10 mmHg]	–1.0 (–3.6 to + 1.7)	0.471	0.506
Resting heart rate [per 10 L/min]	–**5.8 (**–**8.3 to** –**3.2)**	** < 0.001**	0.142
eGFR [per 10 mL/min/1.73 m^2^]	–**3.4 (**–**5.5 to** –**1.3)**	**0.001**	0.491
Hemoglobin [per g/dL]	–0.7 (–3.4 to + 2.0)	0.607	0.071
NT-proBNP [per log pg/mL]	–**4.5 (**–**7.9 to** –**1.2)**	**0.008**	0.349
Body Mass Index [per kg/m^2^]	–**4.8 (**–**5.4 to** –**4.3)**	** < 0.001**	0.784
LDL [per 10 mg/dL]	** + 0.9 (+ 0.1 to + 1.6)**	**0.030**	0.088
HDL [per 10 mg/dL]	** + 7.5 (+ 5.8 to + 9.1)**	** < 0.001**	0.063
Triglycerides [per 10 mg/dL]			
Women Men	–**1.8 (**–**2.5 to** –**1.1)** –**0.9 (**–**1.3 to** –**0.5)**	** < 0.001** ** < 0.001**	0.018
HbA1c [per 0.1%]	–**1.9 (**–**2.4 to** –**1.4)**	** < 0.001**	0.792
Fasting glucose [per mmol/L]	–**8.4 (**–**11.2 to** –**5.6)**	** < 0.001**	0.754
LAVi [per 10 mL]	–0.9 (–4.5 to + 2.8)	0.638	0.819
LVEDVi [per 10 mL]	** + 4.8 (+ 2.2 to + 7.5)**	** < 0.001**	0.928
LVMi [per 10 g]	–1.0 (–2.7 bis + 0.6)	0.227	0.061
LV ejection fraction [per 10%]			
Women Men	** + 7.7 (+ 0.4 bis + 15.0)** ** + 19.1 (+ 11.7 bis + 26.5)**	**0.038** ** < 0.001**	0.031
LV cardiac output [per 100 mL]	-0.2 (-0.5 bis + 0.1)	0.139	0.164
LV stroke volume [per 10 mL]			
Women Men	–1.5 (–4.9 bis + 1.9) ** + 3.1 (+ 0.5 bis + 5.6)**	0.395 **0.020**	0.035
e’_**average**_ [cm/s]	** + 2.0 (+ 7.2 bis + 3.4)**	**0.002**	0.869
E/e’_**average**_	–**3.3 (**–**4.6 bis** –**2.1)**	** < 0.001**	0.082

All entries in bold highlight significant values

*eGFR* estimated glomerular filtration rate, *NT-proBNP* N-terminal pro B-type natriuretic peptide, *LDL* low-density lipoprotein, *HDL* high-density lipoprotein, *HbA1c* hemoglobin A1c, *LAVi* left atrial volume index, *LVEDVi* left ventricular end diastolic volume index, *LVMi* left ventricular muscle mass index, *LVEF* left ventricular ejection fraction, *e'* early myocardial relaxation velocity, *E* early mitral valve inflow velocity

### Reference values of the 6MWD

Six hundred eighty-one participants with valid 6MWD (25%; 60% women, 52 ± 10 years) were considered apparently healthy (Table 1). Their mean 6MWD was 578 ± 71 m. Blood pressure before and after the 6MWT were 122 ± 12/74 ± 8 mmHg and 150 ± 20/82 ± 9 mmHg, respectively. Perceived dyspnea were 0.0 before and 0.7 immediately after the 6MWT; the perceived exertion immediately after the 6MWT was 9.0.

Based on the fact, that the 6MWD was associated with age (with a significant change in slope at the age of 56.2 years) and body height but not with sex, we derived age- and height-specific reference percentiles from these apparently healthy participants (Table 4).

**Table 4:** Reference percentiles of the 6-min walk distance

Body height	Percentile	40 years	50 years	60 years	70 years	80 years
150 cm	2.5	436	434	414	364	313
	10	470	468	448	397	347
	25	511	509	489	439	388
	*50*	*555*	*553*	*533*	*483*	*432*
	75	592	590	570	520	469
	90	631	629	609	558	508
	97.5	679	677	656	606	556
160 cm	2.5	455	453	433	382	332
	10	489	487	466	416	366
	25	530	528	508	457	407
	*50*	*574*	*572*	*552*	*501*	*451*
	75	611	609	588	538	488
	90	650	648	627	577	526
	97.5	697	695	675	625	574
170 cm	2.5	474	472	451	401	351
	10	507	505	485	435	384
	25	549	546	526	476	425
	*50*	*593*	*590*	*570*	*520*	*469*
	75	629	627	607	557	506
	90	668	666	646	595	545
	97.5	716	714	694	643	593
180 cm	2.5	492	490	470	419	369
	10	526	524	503	453	403
	25	567	565	545	494	444
	*50*	*611*	*609*	*589*	*538*	*488*
	75	648	646	626	575	525
	90	687	685	664	614	564
	97.5	735	733	712	662	611
190 cm	2.5	511	509	488	438	388
	10	544	542	522	472	421
	25	586	584	563	513	463
	*50*	*630*	*628*	*607*	*557*	*507*
	75	667	665	644	594	544
	90	705	703	683	633	582
	97.5	753	751	731	680	630

We used the following regression equation:

$$\boldsymbol P\boldsymbol r\boldsymbol e\boldsymbol d\boldsymbol i\boldsymbol c\boldsymbol t\boldsymbol e\boldsymbol d\boldsymbol{\mathit\;}\boldsymbol6\boldsymbol M\boldsymbol W\boldsymbol D=592.134+0.203\times\left(\mathrm{age}<56.2\right)\times\left(56.2-\mathrm{age}\right)-5.034\times(\mathrm{age}>56.2)\times(\mathrm{age}-56.2)+1.857\times(\mathrm{height}-172.6)$$

An example for linear interpolation is given in the supplement. We further provide a Web-based tool for online calculation (https://6mwt.org/).

### Quality assurance

*N* = 11 volunteers (32 ± 8 years, 8 women) performed serial 6MWT. The mean 6MWD of the first 6MWT using the 15-m test distance was 727 ± 57 m, of the second 6MWT using the 15-m test distance was 755 ± 71 m, and of the 6MWT using the 30-m test distance was 800 ± 78 m, respectively. Hence, the mean difference between the first and the second 15-m 6MWT was 28 ± 30 (95%CI 8; 48) m; the mean difference between the second 15-m 6MWT and the 30-m 6MWT was 44 ± 29 (95%CI 25; 64) m.

## Discussion

In a well-characterized population-based sample, the 6MWD was significantly associated with age and body height, but not with sex. Accordingly, we derived age- and height-specific reference percentiles and provided a table to be used by linear interpolation. To enhance practical usage, we also provided an online calculator to determine an individual’s predicted 6MWD, which allows to put the effective 6MWD in relation to an expected result. In addition, we display the effect estimates of further determinants of the 6MWD in the general population like body mass index, metabolic parameters, and cardiac structure and function, to facilitate the refinement of an individual’s predicted 6MWD. These data might serve to grade an individual’s physical capacity and form a good basis for further research in patient collectives.

There is a large body of evidence that the 6MWT is a valid and reliable test with good construct validity and low test–retest variability [16]. Further, the 6MWD shows a strong relationship with measures of exercise performance as assessed for example by cardio-pulmonary exercise testing (CPET) [5, 16]. Although there is a good correlation with maximal oxygen uptake in CPET, the 6MWT is considered a submaximal exercise test, which better reflects an individual’s physical performance in daily life [5]. As detailed in a consensus document from the European Respiratory Society and the American Thoracic Society, the 6MWD is strongly associated with the risk of rehospitalization and mortality in patients with respiratory diseases. Further, the 6MWD is responsive to treatment effects and a *minimal important difference* of 30 m has been suggested as a meaningful change in patients with chronic respiratory diseases [16]. In patients with heart failure, the 6MWD also has been shown to carry prognostic information in the event of an acute decompensation as well as in the chronic state [5]. A recent meta-analysis reported that an increase in 6MWD of 80 m was associated with an improvement in quality of life in patients with heart failure [5, 30].

Nevertheless, the 6MWT is sensitive to changes in methodology. When compared to a straight indoor hallway, participants using a treadmill achieved a shorter 6MWD. By contrast, individuals achieved longer 6MWD when using a continuous (oval or circular) track or an outside track [16, 31]. The impact of the length of the respective test track on the 6MWD has been discussed controversially. In patients with chronic obstructive pulmonary disease [32], there was a difference in 6MWD depending on the length of the test track, there was no such effect observed in patients with severe pulmonary emphysema [31].

Further, there is a learning effect with longer 6MWD achieved during repeat performances of the 6MWT. This effect has been described in different populations, but with markedly varying orders of magnitude [16]. In patients with chronic respiratory diseases, the increase in the 6MWD ranged between 24 and 29 m, with 50–87% of patients walking a larger distance at the second occasion of the 6MWT [16].

Integrating current knowledge, the authors of the abovementioned consensus paper [16] recommend using a test distance of at least 30-m length and to perform the 6MWT twice as a baseline if serial assessment is planned. Nevertheless, both recommendations impose major challenges in clinical practice and several methodologies including varying test distances have been applied [5, 16]. Our quality assurance measures confirmed a longer 6MWD when performing the 6MWT a second time as well as a longer 6MWD when using a 30-m corridor compared to a 15-m test distance. Both findings have to be taken into consideration, when evaluating an individual’s 6MWD and highlight the need for standardization of methodology.

The 6MWD can be used to assess an individual’s physical performance. A prerequisite for valid grading of the 6MWD are reference values the effective 6MWD can be compared with. Several reference equations have been reported that were derived from collectives of different size, age, and sex distribution, using heterogeneous methodology [4, 5, 16, 33]. The application of these equations to a large set of patients with COPD resulted in largely varying and significantly differing values for the predicted 6MWD [4]. Hence, there is consensus that one should apply reference values from a matching population and with identical operating procedures and methodological standards.

Further, previous work reported a significant impact of age on 6MWD as well as of sex, height, weight, and other factors. To our knowledge, the studies identifying sex as significant determinant of the 6MWD did not test for sex interaction and did not adjust sex for height [4, 33]. When doing so, we found height but not sex a significant determinant of the 6MWD. Hence, we provide reference values adjusted for age and height as non-modifiable characteristics. We further provide estimates of the impact of other influencing factors for further refinement of an individual’s predicted 6MWD. An example of linear interpolation is shown in the appendix.

Higher body mass index and adverse metabolic profile as well as higher resting heart rate were associated with shorter 6MWD while more favorable cardiac structure and function went along with longer 6MWD. The negative association of eGFR with 6MWD in this collective of individuals with normal kidney function might be explained by higher muscle mass resulting in lower eGFR based on the underlying equation [34]. Nevertheless, in this population-based sample without symptomatic heart failure, we also found higher NT-proBNP associated with shorter 6MWD indicating myocardial stress associated with lower physical performance. The respective association has to be explored further in symptomatic patient collectives.

We here provide reference percentiles which can be applied to individuals aged 30 to 85 years who performed the 6MWT in a straight and flat 15-m indoor test walk. It allows to compare an individual’s test performance to the 6MWD of a healthy person of similar age and height. The 15-m setup is likely to facilitate testing as it is more readily available in doctors' offices as well as in patients’ homes. Screening and even repetitive assessment of physical performance in patients with chronic diseases including symptomatic heart failure might prove helpful in clinical decision making, but might also contribute to self-empowerment and self-motivation in these patients. Finally, the utilization of the online calculator facilitates this process and visualizes the test result. Translating the tool into a smartphone application might increase its acceptance not only in the population at large, but also in diseased groups of patients.

## Conclusion

In a well-characterized representative sample of the general population, we identified age and body height, but not sex, independent determinants of the 6-min walk distance. Hence, we calculated age- and height-specific reference percentiles, which can be applied to individuals aged 30 to 85 years who performed the 6-min walk test in a straight and flat 15-m indoor test walk. We provide an online calculator to determine an individual’s predicted 6MWD. In addition, we display the effect estimates of further determinants of the 6MWD in the general population. Taken together, our results allow putting an individual person’s effective 6MWD in relation to an expected result.

## Supplementary Information

Below is the link to the electronic supplementary material.

Supplementary file1 (DOCX 583 KB)

## Data Availability

Data can be made available upon request.
