# Supplementary material for: Determinants and reference values of the 6-min walk distance in the general population—results of the population-based STAAB cohort study
Source: Clin Res Cardiol. 2024 Jan 18;114(9):1098–108. doi: 10.1007/s00392-023-02373-3 (PMC12408663; doi:10.1007/s00392-023-02373-3)
Supplement: Supplementary file 1 — Supplementary file1 (DOCX 583 KB) [file 392_2023_2373_MOESM1_ESM.docx]

**Determinants and reference values of the six-minute walk distance in the general population – Results of the population-based STAAB cohort study**

Caroline Morbach, Nicola Moser, Vladimir Cejka, Michael Stach, Floran Sahiti, Fabian Kerwagen, Stefan Frantz, Rüdiger Pryss, Götz Gelbrich, Peter U Heuschmann^*^, Stefan Störk^*^

on behalf of the STAAB consortium

* these authors contributed equally

**Appendix - Supplementary Material**

**Content**

**Supplementary table page**

**Table e1:** example of linear interpolation 2

**Supplementary figures**

**Figure e1**: association of the six-minute walk distance (6MWD) with age 3

**Figure e2**: association of six-minute walk distance (6MWD) with age and sex in 4 individuals A) smaller and B) equal or larger than 171cm body height.

**Supplementary tables**

**Table e1:** example of linear interpolation

Percentiles for interim values of age and body height can be calculated using linear interpolation. Given an individual of 67 years of age and 162 cm body height: 67 years is 70:30 on the distance 60 to 70 years, hence we weight the 60 years with 0.3 and the 70 years with 0.7. 162 cm is 20:80 on the distance 160 to 170 cm, hence we weight the 160 cm with 0.8 and 170 cm with 0.2. Table e1 shows the weighting of the respective combinations of age and body height:

| **Body height** [cm] | **Weighting of body height** | **Age** [years] | **Weighting of age** | **Total weighting of the respective combination of height and age** | **10^th^ percentile of 6MWD** [m] **of the respective combination of height and age** |
| --- | --- | --- | --- | --- | --- |
| 160 | **0.8** | 60 | **0.3** | **0.24** | 466 |
| 160 | **0.8** | 70 | **0.7** | **0.56** | 416 |
| 170 | **0.2** | 60 | **0.3** | **0.06** | 485 |
| 170 | **0.2** | 70 | **0.7** | **0.14** | 435 |

The linear interpolated 10th percentile of the 6-minute walk distance (6MWD) of the respective individual can be calculated based on the reference percentiles given in table 4 as follows: 0,24*466 m + 0,56*416 m + 0,06*485 m + 0,14*435 m = **435 m**

**Supplementary figures**

**Figure e1**: association of the six-minute walk distance (6MWD) with age.

**Figure e2**: association of six-minute walk distance (6MWD) with age and sex in individuals A) smaller and B) equal or larger than 171cm body height.
